# Repurposing Carbamazepine To Treat Gonococcal Infection in Women: Oral Delivery for Control of Epilepsy Generates Therapeutically Effective Levels in Vaginal Secretions

**DOI:** 10.1128/aac.00968-22

**Published:** 2023-01-05

**Authors:** Lucy K. Shewell, Christopher J. Day, Xavier De Bisscop, Jennifer L. Edwards, Michael P. Jennings

**Affiliations:** a Institute for Glycomics, Griffith University, Gold Coast, Queensland, Australia; b The Center for Microbial Pathogenesis, The Abigail Wexner Research Institute at Nationwide Children's Hospital, Columbus, Ohio, USA; c The Department of Pediatrics, The Ohio State University College of Medicine, Columbus, Ohio, USA

**Keywords:** *Neisseria gonorrhoeae*, antimicrobial agents, carbamazepine, drug repurposing

## Abstract

Neisseria gonorrhoeae has developed resistance to all previous antibiotics used for treatment. This highlights a crucial need for novel antimicrobials to treat gonococcal infections. We previously showed that carbamazepine (Cz), one of the most commonly prescribed antiepileptic drugs, can block the interaction between gonococcal pili and the I-domain region of human complement receptor 3 (CR3)—an interaction that is vital for infection of the female cervix. We also show that Cz can completely clear an established N. gonorrhoeae infection of primary human cervical cells. In this study, we quantified Cz in serum, saliva, and vaginal fluid collected from 16 women who were, or were not, regularly taking Cz. We detected Cz in lower reproductive tract mucosal secretions in the test group (women taking Cz) at potentially therapeutic levels using a competitive ELISA. Furthermore, we found that Cz concentrations present in vaginal fluid from women taking this drug were sufficient to result in a greater than 99% reduction (within 24 h) in the number of viable gonococci recovered from *ex vivo*, human, primary cervical cell infections. These data provide strong support for the further development of Cz as a novel, host-targeted therapy to treat gonococcal cervicitis.

## INTRODUCTION

Antimicrobial resistance in Neisseria gonorrhoeae, the causative agent of the sexually transmitted disease gonorrhea, is recognized as an urgent global health threat by the World Health Organization (WHO) and the Centers for Disease Control and Prevention (CDC) (1–3). N. gonorrhoeae has developed resistance to all previous antibiotics recommended for gonorrhea treatment (2). The last remaining treatment option for infections caused by this pathogen was the injectable, extended-spectrum cephalosporin, ceftriaxone, used in combination with azithromycin (4). However, with reports from all over the world of N. gonorrhoeae strains that are resistant to azithromycin (5–10), the CDC recently updated its recommendation for gonorrhea treatment to comprise a single intramuscular dose of ceftriaxone only. Gonococcal infections are asymptomatic in up to 80% of women, but infection can result in serious sequelae, including pelvic inflammatory disease, ectopic pregnancy, infertility, and blindness in neonates. Furthermore, co-infection with N. gonorrhoeae can enhance the transmission of HIV between sex partners (11). The lack of an effective vaccine, in combination with the rise in multidrug- and extensively drug-resistant strains, highlights the urgent need to identify new drugs to treat gonococcal infections.

Complement receptor 3 (CR3, also known as Mac-1, CD11b/CD18, integrin α_M_β_2_) is a member of the β_2_ integrin family (12, 13) and is used by numerous human pathogens to initiate infection and/or promote disease. CR3 is expressed primarily on leukocytes, but it is also expressed on cervical (14) and, potentially, on rectal epithelial cells (15). Though the primary ligand of CR3 is iC3b, the inactive cleavage product of complement protein C3b, CR3 is a promiscuous receptor that can interact with a wide variety of ligands (16). The alpha subunit (CD11b) of CR3 contains an ~200-amino acid insertion domain, or I-domain, that is partly responsible for its multiligand binding properties (17). The interaction between CR3 and N. gonorrhoeae occurs via the I-domain (18) and is mediated by bacterial pili (long filamentous adhesins), with the pilin-linked glycans playing a crucial role (19). Although all pilin-linked glycan structures are capable of binding to the CR3 I-domain, the binding affinity for each of these interactions differs substantively (20). As the specific pilin-linked glycan structure expressed by gonococci is prone to phase variation (random on/off switching of gene or protein expression), any given population of gonococci can express a mixture of pilin-linked glycan structures that terminate in galactose (predominately, *pglA* “on”), diacetylbacillosamine (*pglA* “off”), and/or glucose (*pglH* “on,” in the subgroup of strains that have this gene).

Previously, we used surface plasmon resonance (SPR) to screen a library consisting of 3,141 FDA-approved compounds for their ability to bind to human CD11b recombinant I-domain (rI-domain). During this screen, we identified carbamazepine (Cz) as one compound that bound with high-affinity to rI-domain and that could block the interaction of gonococcal pilin with human rI-domain and with the CR3 protein, *in vitro* and *ex vivo* (20). Using a panel of 11 N. gonorrhoeae strains, including multidrug-resistant and extensively drug-resistant isolates, we found that a single dose (10 μM), 48-h treatment with Cz could completely clear an established N. gonorrhoeae infection of human, primary cervical epithelial (Pex) cells with no development of resistance (20).

Cz is one of the most widely prescribed treatments for epileptic seizures, and is sold under various trade names, including Tegretol, Epitol, and Carbamazepine Sandoz (21). Since the 1960s, Cz has been prescribed worldwide as a first-line treatment for focal onset seizures, but it is also used to treat bipolar disorder as well as to relieve pain in trigeminal neuralgia (22). Currently, Cz is available as an oral preparation (23). Dosage usually starts at 200 to 400 mg/day but can be increased to 800 to 1,600 mg/day (15 to 20 mg/kg/day). As it relates to its current indication, the therapeutic range of Cz in plasma is 4 μg/mL to 12 μg/mL (16.9 μM to 50.7 μM) (24). However, for Cz to effectively treat N. gonorrhoeae, it would need to be present at anatomical sites of infection, the most common of which is the urogenital tract. To our knowledge, no study has previously investigated Cz levels in vaginal secretions following oral dosing. Therefore, this study investigated whether Cz, taken orally, can be detected in vaginal fluid at therapeutically effective levels sufficient to prevent and treat gonococcal infections in women.

## RESULTS

### Measurement of Cz levels in serum.

We collected serum, saliva, and vaginal fluid from six women currently taking Cz (test group) and from 10 women who had never taken Cz (control group). We used a commercial pathology service to measure Cz in all participant serum samples (Table 1) (two independent analyses). In women not taking Cz (control group), Cz was below the reportable range of the assay (2.0 to 20.0 μg/mL), whereas serum from all six test subjects (women taking Cz) had detectable Cz serum levels ranging from mean values of 7.15 μg/mL to 11.95 μg/mL (30.26 μM −50.58 μM). There was agreement between the Cz dosage of the test group and the serum concentrations obtained, with the participant taking the highest dose of Cz (CBP12, 1200 mg/day) returning the highest serum concentration (11.95 μg/mL) and the participant taking the lowest dose (CBP14, 200 mg/day) returning the lowest serum concentration (7.15 μg/mL).

**TABLE 1:** Participant information and Cz concentrations in bodily fluids determined in this study^*a*^

PN	Cz dosage/other medications	Age	Serum (CP)	Serum (ELISA)	Saliva (ELISA)	V. fluid (ELISA)
CBP01	n.t.^*b*^	40	<2.0 μg/mL	<0.244 μg/mL	<0.244 μg/mL	<0.244 μg/mL
CBP02	n.t.	19	<2.0 μg/mL	<0.244 μg/mL	<0.244 μg/mL	<0.244 μg/mL
CBP03	n.t.	31	<2.0 μg/mL	<0.244 μg/mL	<0.244 μg/mL	<0.491 μg/mL
CBP04	n.t.	30	<2.0 μg/mL	<0.244 μg/mL	<0.244 μg/mL	<0.244 μg/mL
CBP05	n.t.	23	<2.0 μg/mL	<0.244 μg/mL	<0.244 μg/mL	<0.244 μg/mL
CBP06	n.t.	31	<2.0 μg/mL	<0.244 μg/mL	<0.244 μg/mL	<0.244 μg/mL
CBP07	n.t.	32	<2.0 μg/mL	<0.244 μg/mL	<0.244 μg/mL	<0.244 μg/mL
CBP08	400 mg/day	28	11.3 μg/mL (1.13)	9.91 μg/mL (0.862)	6.349 μg/mL (0.168)	8.453 μg/mL (0.353)
CBP09	n.t.	30	<2.0 μg/mL	<0.244 μg/mL	<0.244 μg/mL	<0.244 μg/mL
CBP10	n.t	21	<2.0 μg/mL	<0.244 μg/mL	<0.244 μg/mL	<0.244 μg/mL
CBP11	n.t.	30	<2.0 μg/mL	<0.244 μg/mL	<0.244 μg/mL	<0.244 μg/mL
CBP12	1200 mg/day	19	11.95 μg/mL (1.34)	11.35 μg/mL (0.073)	6.597 μg/mL (0.243)	6.903 μg/mL (0.332)
CBP13	400 mg/day; also taking Epilim^*c*^ 200 mg/day	46	7.8 μg/mL (0.707)	7.59 μg/mL (0.322)	4.364 μg/mL (0.747)	1.148 μg/mL (0.243)
CBP14	200 mg/day; also taking Keppra^*d*^ 1 g/day	40	7.15 μg/mL (0.495)	6.79 μg/mL (0.164)	4.801 μg/mL (0.419)	4.692 μg/mL (0.408)
CBP15	400 mg/day; also taking Keppra 1 g/day	20	9.2 μg/mL (0.566)	12.24 μg/mL (1.51)	5.729 μg/mL (0.655)	6.385 μg/mL (0.260)
CBP16	800 mg/day; also taking Keppra 1.5 g/day	48	11.35 μg/mL (1.06)	11.68 μg/mL (0.983)	5.287 μg/mL (0.555)	8.751 μg/mL (0.062)

a Test and control sera were sent to a commercial pathology service (CP) on two independent occasions for measurement of Cz levels. Cz concentrations in serum were also determined using a competitive ELISA developed in this study. Cz concentrations in serum are reported as the mean of duplicate, independent analyses as determined by the commercial pathology service and as the mean of triplicate wells determined using the competitive ELISA. Cz concentrations in saliva and in vaginal fluid were determined using the competitive ELISA, and data are shown as the mean of duplicate, independent assays (each performed in triplicate). For samples in which measurable Cz was detected, the standard deviation is provided in parentheses. PN: Participant number. <2.0 μg/mL = less than the reported range of the immunoassay performed by the CP (2.0 to 20.0 μg/mL). <0.244 μg/mL = less than the lower limit of quantification (LLOQ) of the competitive ELISAs.

b n.t. = not taking the drug (control participants).

c Epilim is the trade name for sodium valproate, an anti-convulsant drug (54).

d Keppra is the trade name for levetiracetam, an anti-convulsant drug (55).

### Measurement of Cz levels in saliva and vaginal fluid using competitive ELISA.

**FIG 1:**
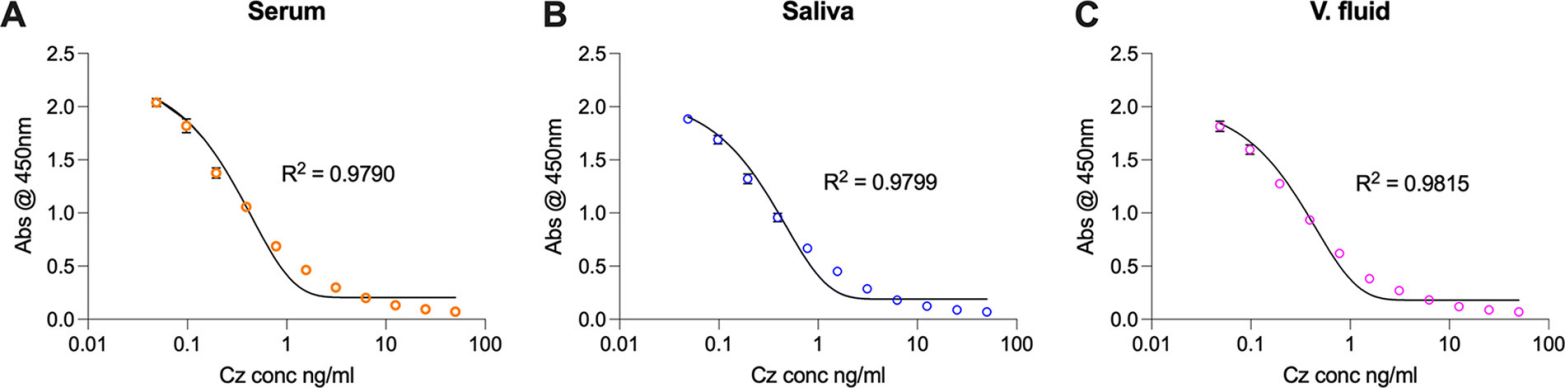
Representative standard curves from Cz competitive ELISAs. Cz was spiked into a 1:5,000 dilution of (A) normal human serum (NHS), (B) control saliva, or (C) control vaginal fluid starting at 50 ng/mL, then 2-fold serially diluted with a 1:5,000 dilution of (A) NHS, (B) control saliva, or (C) control vaginal fluid to yield a test range of 0.0488 ng/mL to 50 ng/mL (equivalent to 0.244 μg/mL up to 250 μg/mL in 100% serum/saliva/fluid). The last well of each row of the microtiter plate was left as the diluent only control. Data are shown as the mean of triplicate wells, +/–1 SD, from one assay. The line of best fit was generated using a sigmoidal, 4-parameter logistic, X = log(concentration) model using GraphPad Prism 9.

We were unable to find a commercial pathology service to measure Cz levels in saliva or in vaginal fluid; therefore, we adapted a competitive ELISA to measure Cz concentrations in these bodily fluids. A competitive ELISA is based upon competition between a labeled and an unlabeled analyte, or ligand, in a sample for a limited number of antibody binding sites bound to the wells of a microtiter plate. In this assay format, the absorbance is inversely proportional to the concentration of analyte, Cz in this case, in the sample. To measure Cz in our participant samples, wells were coated with an anti-Cz polyclonal antibody. Cz standard curves were established by spiking Cz into either normal human serum, control saliva, or control vaginal fluid that was then serially diluted 2-fold, as described in Materials and Methods (Fig. 1). Irrespective of the diluent used to prepare each standard, the lower limit of quantification (LLOQ) for each assay was 0.0488 ng/mL (207 pM, the lowest concentration standard used), which is equivalent to 0.244 μg/mL (1.03 μM) in 100% serum/saliva/vaginal fluid.

We assayed all test and control serum samples using the competitive ELISA (Table 1). Cz was below the LLOQ for all control serum samples. Serum Cz levels determined for the test subjects ranged from 6.79 μg/mL to 12.24 μg/mL (28.74 μM to 51.81 μM) (Table 1). Four out of six test subjects had Cz levels within 1 standard deviation (SD) of the corresponding value reported by the commercial pathology service. For participant CBP08, the level of Cz determined by the competitive ELISA was within 2 SDs of that determined by the commercial pathology service. We recorded higher (12.24 μg/mL +/– 1.51 μg/mL) Cz levels than the commercial pathology service (9.2 μg/mL +/– 0.566 μg/mL) for participant CBP15. The Cz levels determined by the competitive ELISA for five out of the six test subjects were within 80% to 120% of the Cz levels determined by the commercial provider (Table S1). The overall accuracy of the competitive ELISA compared to the commercial provider was 102% (Table S1).

We then assayed the saliva and vaginal fluid collected from test and control subjects in the competitive ELISA (Table 1). Samples were diluted 1:5,000, 1:10,000, and 1:20,000, and the highest concentration dilution that fell within the linear range of the standard curve was used to interpolate the Cz concentration in each specimen. All control group saliva samples were found to have Cz levels below the assay LLOQ. The range of Cz concentrations in saliva samples from the test group was 4.36 μg/mL to 6.60 μg/mL (18.45 μM to 27.94 μM) (Table 1). There was a significant correlation (Pearson *r* = 0.8229; *P*-value = 0.0443) between mean Cz concentrations determined by ELISA for saliva and the mean serum Cz concentrations determined by the commercial pathology laboratory (test subjects only). Linear regression showed that serum Cz levels significantly predicted saliva Cz levels (R^2^ = 0.6772; *P*-value = 0.0443) (Fig. S1A). This was expected based on previous reports of a linear relationship between serum and saliva Cz concentrations (25–27).

All control group vaginal fluid samples, except the specimen from CBP03, had Cz concentrations below the LLOQ of the assay, whereas the Cz concentrations for the test group ranged from 1.15 μg/mL to 8.75 μg/mL (4.87 μM to 37.04 μM). The concentration of Cz in vaginal fluid from control participant CBP03 was <0.491 μg/mL (<0.244 μg/mL in assay 1 [below LLOQ], 0.738 μg/mL in assay 2); thus, although analysis of this specimen resulted in detectable Cz (in 1 experiment), the absorbance values recorded for CBP03 were not in the linear range of the standard curve. Therefore, the concentration determined may not be accurate and may be the result of increased background attributed to human error and/or interassay variability. Although higher vaginal fluid Cz concentrations were associated with higher serum Cz levels, there was not a significant correlation (Pearson *r* = 0.7963; *P*-value = 0.0580), nor did linear regression show that serum Cz levels significantly predicted Cz levels in vaginal fluid (R^2^ = 0.6341; *P*-value = 0.0580) (Fig. S1B). We believe that this is the first report to show that Cz, taken orally, can be found in vaginal mucosal fluid at concentrations that may be therapeutically effective in preventing and treating gonorrhea.

### Vaginal fluid from women taking carbamazepine can cure a N. gonorrhoeae infection of Pex cells, an *ex vivo* model of cervicitis.

**FIG 2:**
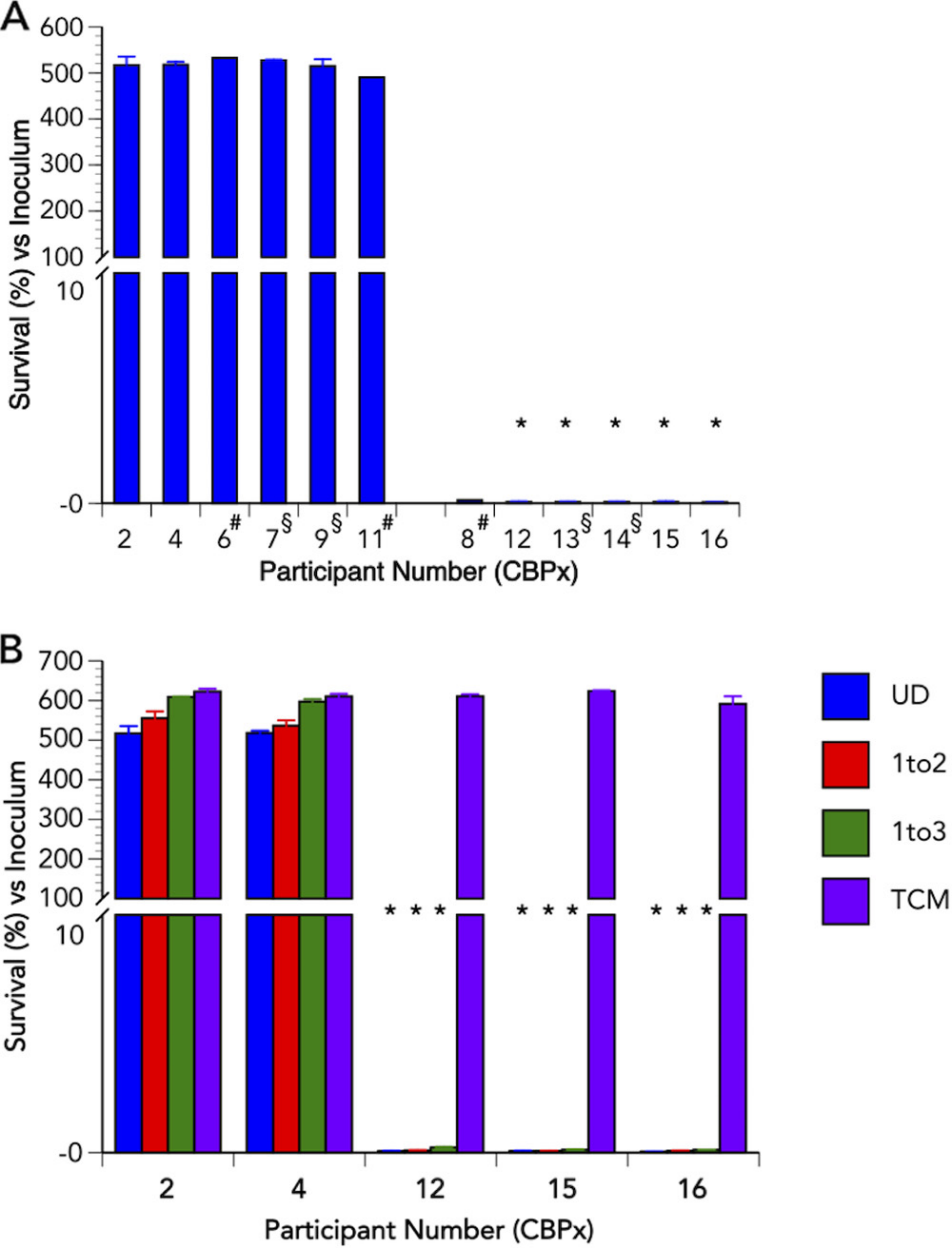
Carbamazepine is present in vaginal fluid at concentrations sufficient to effectively treat gonococcal infection. The effect of Cz on N. gonorrhoeae strain WHO Z survival during an established Pex cell infection was examined using vaginal fluid from participants (CBP) whom were either not taking (participants CBP02, 04, 06, 07, 09, and 11) or taking (participants CBP08, 12, 13, 14, 15, and 16) Cz for medically indicated reasons not related to the present study. Values given are the mean (variance) of the percentage of viable gonococci recovered from Pex cells at 24 h posttreatment versus the infection inocula. (A) Data are shown for all study participants, in which assays were performed using undiluted (10%) vaginal fluid. Limited sample availability only allowed for an n of 1 (#) or an n of 2 (§) for some participant specimens, as noted on the *x* axis. Samples from all other participants (CBP02, 04, 12, 15, and 16) were examined on three separate occasions. (B) Experiments were performed using undiluted (UD, 10%) vaginal fluid, vaginal fluid that was diluted 1:2 (5%) or 1:3 (3.3%) in tissue culture medium, or in tissue culture medium (TCM), as noted. Experiments were performed on three separate occasions using vaginal fluid from CBP02, 04, 12, 15, and 16. *, *P ≤ *0.0196 versus infections performed in tissue culture medium. The percentage of viable gonococci recovered from Pex cell infections is provided in Table S2. Data for those study participants (CBP06, 07, 09, 11, 08, 13, and 14) in which the volume of available vaginal fluid was limited and only allowed for an n of 1 (#) or an n of 2 (§) are shown in Fig. S2.

To assess the ability of Cz in vaginal fluid to cure a N. gonorrhoeae infection in a biological system, we performed infection “cure” assays under physiological (37°C, 3% O_2_) conditions using primary human cervical epithelial (i.e., Pex) cells. All specimens were assayed at a final 1:10 dilution in PBS (i.e., 10% of the *in vivo* vaginal fluid concentration), or were further diluted to 5% or 3.3% of the *in vivo* concentration. When compared to parallel assays performed using tissue culture medium only (control, no vaginal fluid), the number of viable gonococci recovered from Pex cell infections that were treated for 24 h with 10% vaginal fluid from test participants was significantly (*P ≤ *0.0196) reduced more than 99.9% (Fig. 2A; Fig. S2; Table S2). Cz in 5% or 3.3% vaginal fluid (test) specimens was equally effective in treating Pex cell infections as was 10% vaginal fluid (Fig. 2B; Fig. S2; Table S2). Data obtained using control specimens, i.e., from women not taking Cz, indicated that vaginal fluid by itself had a marginal, but significant (*P ≤ *0.0164), effect on N. gonorrhoeae viability (Fig. 2B; Fig. S2; Table S2). In this regard, the 10% control specimens reduced the number of viable gonococci recovered from Pex cells by ≤ 20.6%, an effect which was less pronounced with the use of 5% or 3.3% control group specimens (Table S2). Collectively, these data indicate that the concentration of Cz found within the vaginal fluid of women taking Cz, which is ≥10-fold higher than the samples tested in these assays, is sufficient to cure *ex vivo* cervical infection by N. gonorrhoeae.

**FIG 3:**
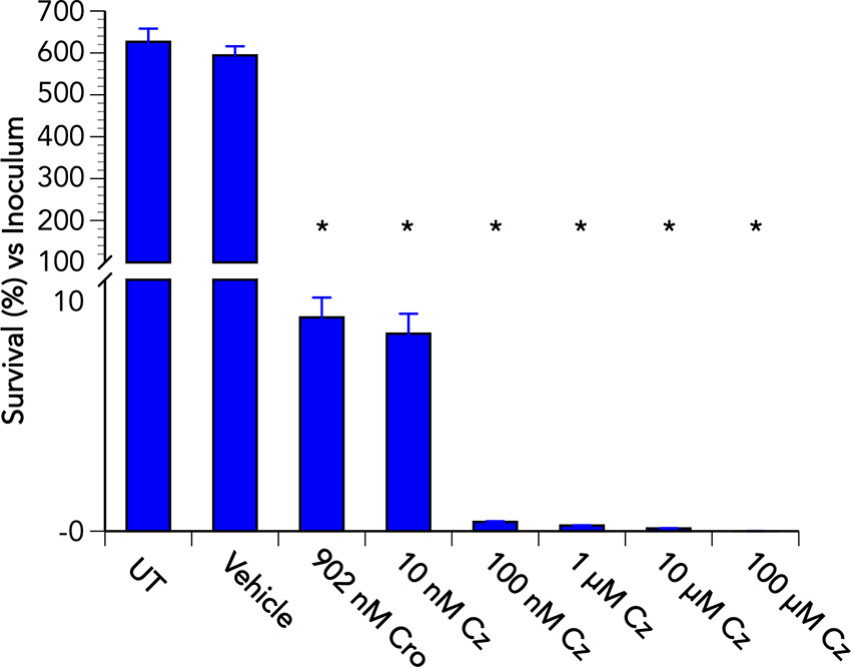
Carbamazepine in vaginal fluid is more effective than ceftriaxone in treating Pex cells infected with a multidrug-resistant strain of N. gonorrhoeae. The ability of the multidrug-resistant N. gonorrhoeae strain WHO Z to survive a dose range (*x* axis) of Cz treatment during Pex cell infection; performed using 10% pooled, control vaginal fluid; was examined, as described in the text. Values given are the mean (variance) of the percentage, versus the infection inocula, of viable gonococci recovered at 24 h from established untreated (UT) Pex cell infections or from established infections in which DMSO (vehicle control), ceftriaxone (Cro), or Cz was added to control vaginal fluid. Data were obtained from three independent trials. *, *P ≤ *0.0001 versus the untreated control.

To further investigate the effectiveness of Cz in vaginal fluid for use as a potential therapeutic agent to treat gonococcal cervicitis, “cure” assays were performed using pooled (10%) vaginal fluid from control participants that was spiked with Cz at a dose range of 10 nM to 100 μM (2.4 ng/mL to 23 μg/mL). When compared to untreated or vehicle-treated infections, 24 h treatment with a single dose (≥l0 nM or ≥2.4 ng/mL) of Cz again resulted in a greater than 99% reduction in the number of viable N. gonorrhoeae recovered from Pex cells (Fig. 3). At doses of ≥100 nM (≥23.6 ng/mL), Cz was more effective in curing infection of Pex cells by the multidrug-resistant gonococcal strain, WHO Z, than was ceftriaxone, the current recommended antibiotic for gonorrhea treatment. Consistent with our previous study (20), assays performed in the absence of Pex cells revealed that the ability of Cz to cure Pex cell infections was independent of a direct bactericidal effect of Cz on N. gonorrhoeae. That is, in the absence of Pex cells, bacterial viability was not significantly (*P* ≥ 0.0823) reduced following a 1-h incubation in control vaginal fluid (10%) spiked with Cz (dose range of 10 nM to 100 μM or 2.4 ng/mL to 23 μg/mL) when compared to untreated bacteria or bacteria that were incubated with the vehicle control (Fig. S3). In contrast, a significant (*P ≤ *0.032) reduction in gonococcal viability was observed following treatment with ceftriaxone (0.5 μg/mL or 902 nM) or gentamicin (100 μg/mL or 209 μM) antibiotic controls (Fig. S3). The low volume of vaginal fluid used in these experiments did not sustain N. gonorrhoeae—no viable gonococci were recovered following a 24-h incubation in the absence of Pex cells under any of the conditions tested (data not shown). Taken together, our data support the further development of Cz as an effective, host-target therapy to treat gonococcal cervicitis.

## DISCUSSION

The rapid emergence of multidrug-resistant and “untreatable” gonococcal strains highlights the need for novel therapeutics to treat N. gonorrhoeae infections. Drug repurposing is a promising strategy to “fast-track” the translation of new antibiotics into the clinic by accelerating the drug discovery process and by expediting the time to market (28, 29). We previously showed that Cz can effectively cure infection of primary human cervical (i.e., Pex) cells challenged with a panel of multidrug-resistant or extensively drug-resistant gonococcal strains without the development of resistance (20). Regardless, successful treatment of a gonococcal infection requires that any antimicrobial be efficiently distributed to all possible infection sites (30).

The lower reproductive tract is the primary infection site for N. gonorrhoeae in women. Thus, to aid in the preclinical development of Cz as an anti-gonorrheal agent, we measured Cz concentrations in the vaginal fluid of women who were or were not taking Cz. Vaginal fluid was collected using self-administered vaginal tampons (VTs), which have been used in numerous studies as an acceptable method for the collection of vaginal specimens (31–35). Self-administered VTs offer several advantages over the collection of vaginal swabs by a medical practitioner in being less invasive, causing less distress and/or discomfort to a participant, and in being a procedure that most women perform routinely each month. We have now presented data to indicate that Cz is present within lower reproductive tract mucosal secretions in women taking this drug for medical indications not related to the present study. Moreover, Cz was present in vaginal fluid at concentrations expected to be therapeutically effective against N. gonorrhoeae, based on our previous published work (20) as well as data presented herein (Fig. 2).

We adapted a competitive ELISA to enable Cz quantification in saliva and vaginal fluid, as commercial immunoassays are optimized for the analysis of serum and plasma. Predominant methods used clinically to measure Cz include: enzyme immunoassays (EIA), high performance liquid chromatography (HPLC), gas-liquid chromatography (GLC), and fluorescence polarization immunoassays (FPIA) (36). We choose to use an immunoassay as this was the method used by the commercial pathology service to measure serum Cz levels. The indicated (neurological) therapeutic range for Cz in saliva is reported to be 1.4 μg/mL to 3.5 μg/mL (27, 37). An extensive review by Liu et al. of the studies that have measured Cz in saliva by various methods (EIA, HPLC, GLC and FPIA) reports Cz concentrations ranging from 0.1 μg/mL to 5.5 μg/mL (36). Our reported range of 4.36 μg/mL to 6.60 μg/mL for the six test subjects in this study is at the upper end of the range reported by Liu et al. (36). However, it should be noted that only analytical separation techniques, such as HPLC, can distinguish Cz from its main metabolite, carbamazepine-10,11-epoxide (Cz-E, discussed further below), and many of the immunoassays used to measure Cz have cross-reactivity with Cz-E (38). In this regard, the use of an alternative anti-Cz antibody that exhibits increased specificity for Cz, versus Cz-E, in the competitive ELISA may help to overcome this problem. The polyclonal anti-Cz antibody we used in this study exhibits 61.4% cross-reactivity with Cz-E and 49.5% cross-reactivity with 10,11-dihydro-10-hydroxycarbamazepine (mono-hydroxy derivative). Thus, antibody cross-reactivity in the competitive ELISA may explain the higher concentrations reported in our study for saliva, as both Cz and its metabolite, Cz-E, may have been detected. Studies are on-going in our laboratory to examine the potential effectiveness of this, as well as other Cz metabolites and analogs, in treating gonococcal infections.

Cz was detected in the vaginal fluid of all test subjects at concentrations of 1.15 μg/mL to 8.75 μg/mL (4.87 μM to 37.04 μM). We did not observe a significant correlation between Cz levels in serum and vaginal fluid; however, this was not unexpected. Cz is reported to exhibit wide inter- and intraindividual pharmacokinetic variability due to differences in genetics, age, absorption, autoinduction, and disease states (39, 40), and we could not find any literature regarding the distribution of Cz into the urogenital tract. Furthermore, polytherapy can impact the pharmacokinetics of Cz. One subject, CBP13, had lower than expected levels of Cz in their vaginal fluid, based on their serum and saliva concentrations. This subject was also taking Epilim, the trade name for sodium valproate, another antiepileptic drug commonly given with Cz. Combination therapy of sodium valproate with Cz is reported to increase the Cz-E level relative to total Cz, likely due to inhibition of the further metabolism of Cz-E (41). Detailed pharmacokinetic studies investigating the distribution of Cz to the urogenital tract will be required in future studies to determine the feasibility of oral Cz to treat vaginal gonococcal infections.

The metabolism of Cz occurs via cytochrome P450 system oxidation in the liver, resulting in the formation of Cz-E. Cz-E can be further metabolized by epoxide hydrolase to form pharmacologically inactive, trans-10,11-dihydroxy-10,11-dihydroxycarbamazpine (trans-Cz-diol), which is eliminated via urine. Although Cz is one of the best-tolerated antiepileptic drugs, the active 10,11-epoxide metabolite (Cz-E) can result in adverse drug reactions, including immune-mediated hypersensitivity, hepatotoxicity, and bone disorders (42). Oxcarbazepine (OXC) is an antiepileptic drug structurally similar to Cz, but unlike Cz, it is not oxidized by the cytochrome P450 system. OXC is rapidly reduced by cytosolic enzymes to 10,11-dihydro-10-hydroxy-carbazepine (mono-hydroxy derivative), the clinically relevant metabolite of OXC, thereby eliminating the production of Cz-E. Thus, OXC is better tolerated and has less potential for adverse drug reactions and drug interactions (43). We specifically recruited women taking Cz (under various trade names) in this study, but, as noted above, future studies are planned to determine if the better-tolerated derivates of Cz (e.g., OXC) exhibit comparable effectiveness, as we have reported for Cz, in treating gonococcal infections.

The present study is limited by the number of participants recruited, as well as by the small volume of vaginal fluid specimens obtained from these donors. Of note, we were unable to perform 48-h infection/treatment studies, as the limited volume (150 μL per assay condition) of vaginal fluid available for analysis did not support the viability of infected Pex cells over a 48-h period. In our previous work, we found that a single dose of Cz resulted in 100% clearance of multiple gonococcal strains by 48 h, but also that bacterial viability was significantly reduced by 24-h post-Cz treatment (20). Thus, data presented herein are consistent with our previous published data and suggest that *in vivo*, with a greater than 24-h exposure of bacteria to Cz, gonococcal clearance most likely would be achieved. How Pex cells are cleared of gonococci following Cz treatment has not yet been explored and will be an avenue of future study. Cz can block the interaction of N. gonorrhoeae with CR3 (20), and this most likely contributes to the ability of Cz to “cure” Pex cell infections. However, Cz is capable of curing established gonococcal infections of Pex cells, indicating that mechanisms other than host receptor blocking contribute to the effectiveness of Cz in curing cervical infections *ex vivo*. Thus, it is our working hypothesis that engagement of CR3 by Cz likely alters CR3-dependent cellular responses that, in turn, either result in gonococci trafficking to an intracellular environment that is not conducive to their survival and/or triggers an innate immune response that similarly is not conducive to survival (either inside or outside the host cell). In this regard, it is well established that differential cellular effects are triggered with CR3 engagement and are dependent upon the specific CR3-ligand interaction (44–47), and we have shown that, depending upon the specific gonococcal pilin-linked glycan-CR3 interaction mediating Pex cell adherence, gonococci either survive and proliferate within Pex cells, or they are killed/do not survive ([19] and unpublished data). However, the contribution of other host responses (e.g., sequestration of essential molecules) triggered with Cz engagement of CR3 in gonococcal clearance cannot be ruled out. Regardless, our data provide strong support for the further development of Cz as a novel, host-targeted therapy to treat gonococcal cervicitis. A host-targeted approach to gonorrhea treatment is unlikely to lead to the development of resistance and, thus, may represent a long-term solution to the growing problem of multidrug-resistant N. gonorrhoeae. Additionally, targeting CR3, using Cz or Cz analogs, may be a viable option to treat other antibiotic-resistant human pathogens that use this receptor to initiate infection or to potentiate disease.

## MATERIALS AND METHODS

### Participant recruitment.

This study was approved by the Griffith University Human Research Ethics Committee (GU Ref No. 2019/639) and was conducted in accordance with the National Health and Medical Research Council’s National Statement on Ethical Conduct in Human Research. Written, informed consent was obtained from all women enrolled in the study. Participants were eligible for recruitment if they were between the ages of 18 to 55 years, as people over the age of 65 have altered levels of Cz clearance when compared to younger adults (48). Exclusion criteria were: postmenopausal status, pregnancy, or suspected pregnancy. All participants were de-identified throughout the study by assignment of a participant number (CBP01 to CBP16). Participant specimens were collected between October 2019 and September 2020.

The test group consisted of six women (age range = 19 to 46 years; mean age = 33.5 ± 12.9 years) who were currently taking, at least, 200 mg/day of Cz (trade names: Tegretol 100, Tegretol CR 200, Tegretol CR 400, and Carbamazepine Sandoz) for the treatment of epilepsy and whom had been doing so for at least 1 month. The control group was comprised of 10 women (age range = 19 to 40 years; mean age = 28.7 ± 6.1 years) who had never taken Cz.

### Participant data and sample collection.

Participants were asked to complete a brief questionnaire to record their age as well as their current dosage of Cz and any other medications taken. All serum, saliva, and vaginal fluid samples were processed, as follows, and stored at −80°C within 2 h of collection. Serum was collected using 8.5 mL serum separation tubes (SST II Advance BD Vacutainer, Becton, Dickenson, and Co., Franklin Lakes, NJ, USA), per the manufacturer’s instructions. Approximately 2.5 mL of saliva was collected by having participants spit into a 50 mL screw-cap tube marked with a red line to indicate the required volume. For vaginal fluid specimens, participants were asked to ensure that they were not menstruating at the time of their appointment, and specimens were collected using self-administered VTs. Each participant was given an opaque, clip-seal bag (for privacy) containing a packaged, commercial brand, regular flow VT with an applicator and a sterile 50 mL screw-cap tube. The study coordinator instructed participants to insert the VT as directed by the manufacturer (instructions provided) and to leave the VT in place for 30 min. After 30 min, participants removed the VT and placed it into the provided 50 mL screw-cap tube. The dry weight of three VTs (without applicator) was recorded and found to be essentially identical (2.68 g); therefore, it was assumed that the dry weight of all VTs within the same packet were the same. Each VT was weighed (wet weight) after self-administration to determine the volume of fluid absorbed (wet weight – dry weight = fluid volume, assuming 1 g = 1 mL). The VT was sterilely transferred to the barrel of a 50 mL syringe to which sterile, 1× PBS was carefully pipetted into the middle of the VT to give a final dilution of 1:4 to 1:10. The VT was then compressed with the plunger of the syringe until all liquid was expelled into a sterile 50 mL tube.

### Analysis of serum Cz concentration using a commercial pathology service.

Frozen serum (1 mL), labeled only with the participant number, was shipped on dry ice to a commercial pathology laboratory (Sullivan Nicolaides Pathology, Bowen Hills, QLD, AUS) to conduct measurement of serum Cz levels. Cz was quantified using an enzyme immunoassay on an ARCHITECT c16000 clinical chemistry analyzer (Abbott, Abbott Park, IL, USA). Two independent measurements of serum Cz were performed.

### Competitive ELISA for quantification of Cz.

Wells of 96-well Maxisorp plates (Nunc, Thermo Fisher Scientific, Rochester, NY, USA) were coated (1 h, room temperature [RT]) with 100 ng of a sheep, polyclonal anti-Cz antibody (MyBioSource, San Diego, CA, USA; Cat no. MBS5302163) in 0.05 M carbonate-bicarbonate coating buffer, pH 9.6. Wells were washed three times with PBS/0.05% Tween 20 (PBS-T). Wells were blocked with 150 μL of 3% bovine serum albumin (BSA) in PBS-T at 4°C overnight (O/N) or at RT for 1 h before washing three times with PBS-T. To establish a standard curve, a 5 mg/mL stock solution of Cz (Sigma-Aldrich, St., Louis, MO USA; Cat no. C4024) in DMSO was diluted to 50 ng/mL with 1× PBS and added to triplicate wells. Cz was then 2-fold serially diluted in PBS to a final concentration of 0.0488 ng/mL (final volume = 100 μL), the last row of each multiwell plate contained PBS only (control). 50 μL of a Cz-HRP conjugate (MyBiosource, Cat no. MBS5303945), diluted 1:500 with PBS, was then added to each well. Dilutions of 1:100 and 1:1,000 were also trialed. Samples were mixed gently with the Cz-HRP-conjugate before incubating (1 h, RT, protected from light). Wells were washed five times with PBS-T before adding 50 μL/well of 1-Step Ultra TMB-ELISA Substrate Solution (Thermo Fisher Scientific, Cat no. 34028) and incubated (RT, 30 min, protected from light). Reactions were stopped by the addition of 50 μL 1N HCl, and absorbance was measured at 450 nm using a Tecan 200 Infinite Pro plate reader (Tecan, Mannedorf, CHE).

### Measurement of Cz concentration in serum, saliva, and vaginal fluid using competitive ELISA.

Wells of 96-well Maxisorp plates were coated, blocked, and washed as described above. Cz was quantified in participant samples using a standard curve derived from 2-fold serial dilutions of Cz (concentration range 50 ng/mL to 0.0488 ng/mL) in triplicate wells. For analysis of Cz in participant serum, a 1:5,000 normal human serum (Merck, Darmstadt, DEU; Cat no. H3667) solution in PBS was used as the diluent. To quantify Cz in saliva and vaginal fluid, control saliva, or vaginal fluid, respectively, were similarly diluted 1:5,000 in PBS and then used as the diluent for the standard curves. Control and test subject serum, saliva, and vaginal fluid samples were mixed with PBS to a final dilution of 1:5,000, 1:10,000, and 1:20,000 for use in competitive ELISAs (considering the initial dilution of vaginal fluid). Diluted participant samples (100 μL) were added to triplicate wells after which 50 μL of the Cz-HRP conjugate, diluted 1:500 with PBS, was added. Samples were mixed by gently pipetting up and down at least six times, before incubation (1 h, RT, protected from light). Wells were subsequently washed five times with PBS-T. Cz was then detected using the 1-Step Ultra TMB-ELISA Substrate Solution, and absorbances were measured at 450 nm using a Tecan 200 Infinite Pro plate reader, as described above. One assay was performed using serum samples to validate the assay by comparison to results obtained from the commercial provider (Table S1); whereas, two independent assays were performed for the saliva and vaginal fluid samples.

### Validation of the in-house competitive ELISA.

The interassay (within) precision of the in-house competitive ELISA for the three matrices (serum, saliva, and vaginal fluid) was determined by calculating the coefficient of variation (CoV) for triplicates of each of the standards used in the standard curve. The CoV for each standard was calculated by dividing the standard deviation for the three replicates by the mean, then multiplying by 100 (Table S3). The intraassay (between) precision was determined for saliva and vaginal fluid assays by calculating the CoV for six replicates for each standard used in the standard curve across two independent assays (Table S3). The limit of detection (LOD) for the in-house competitive ELISA was determined as the lowest concentration in the standard curve that was less than 3SD from the mean of the blank samples (diluent only) (49). With competitive ELISA, the blank or negative samples have the highest absorbance values; therefore, the LOD was determined as the value less than 3 SD from the mean of the blank samples rather than 3 SD above the mean of the blank samples. The lower limit of quantification (LLOQ) for the competitive ELISAs for each of the matrices (serum, saliva, and vaginal fluid) was calculated by determining the lowest concentration on the standard curve that gave an intraassay (within; for serum only as only one assay was performed) or an interassay (between assay; for saliva and vaginal fluid) precision, as defined by the CoV, of <20% (50) and was less than 3 SDs from the mean of the blank samples. The LLOQ was found to be 0.048828 ng/mL (equivalent to 0.244 μg/mL in 100% serum, saliva, or vaginal fluid).

### Data analysis.

A sigmoidal, 4-parameter logistic, X is log(concentration) model, with nonlinear regression, was used to fit the standard curves obtained from competitive ELISAs. The Cz concentrations of participant samples were interpolated from standard curves using the highest concentration that was able to give a reliable Cz concentration. Linear regression analysis was performed using serum Cz levels, determined by the commercial pathology service, as the Y variable and either saliva or vaginal fluid Cz levels, determined by the competitive ELISA, as the X variable for test subjects only. All data analyses were performed using GraphPad Prism 9 (GraphPad Software, San Diego, CA, USA).

### Cell culture and bacteria.

De-identified cervical tissues were obtained from the Cooperative Human Tissue Network (Columbus, OH, USA) and used to procure primary human cervical epithelial (i.e., Pex) cells as described previously (51). In brief, this process involves the outgrowth of epithelial cells from dissected cervical tissue. Tissue explants and Pex cells were maintained using defined keratinocyte serum-free medium (dk-SFM; Gibco, Grand Island, NY, USA).

Infection studies were performed using N. gonorrhoeae strain, WHO Z (also named A8806), a multidrug-resistant strain that was isolated in 2013 from a woman with genital tract gonococcal infection (52, 53). Bacteria were obtained from Public Health England, cultured on GC-IsoVitaleX agar plates, and enumerated spectrophotometrically before their use in infections (multiplicity of infection of 100), as previously described (51). To mimic the cervical microenvironment, both Pex cells and bacteria were cultured (37°C) overnight in a tri-gas incubator under microaerobic (3% O_2_) conditions before their use in infection studies.

### Infection and treatment assays.

Treatment/cure assays were performed using a 48-well microplate format, as we have described (20). Vaginal fluid collected from each participant was diluted in PBS to a final 1:10 dilution, filter sterilized using a 0.2 μm syringe filter, and then further diluted 1:2 (5% vaginal fluid final concentration) or 1:3 (3.3% vaginal fluid final concentration) with antibiotic-free cell culture medium. Following a 90-min challenge of Pex cells with WHO Z to establish infection, infected Pex cells were rinsed. Then, 150 μL of participant vaginal fluid (10%, 5%, or 3.3%) or antibiotic-free cell culture medium (no participant vaginal fluid, control), as noted, was then added to each well. Following a 24-h incubation (3% O_2_, 37°C), Pex cell monolayers were lysed. Serial dilutions of the Pex cell lysates were plated, and viable gonococci were enumerated by counting CFU after a 48-h incubation (37°C, 5% CO_2_). The percentage of viable N. gonorrhoeae recovered from Pex cell lysates was determined as a function of the bacterial inoculum. Assays were performed on three separate occasions when possible. However, the available volume of vaginal fluid from some participants, as noted, was only sufficient to complete one or two experiments.

As an alternative approach to determine the effectiveness of Cz as a potential gonorrheal therapeutic agent, infections were performed as noted above. However, Cz was added to pooled (10%) vaginal fluid from control participants (CBP03-05, CBP07, CBP09, and CBP11) that were not taking Cz and diluted 10-fold (dose range: 10 nM to 100 μM or 2.4 ng/mL to 23 μg/mL) using vaginal fluid (10%) as the diluent. Vaginal fluid, spiked with Cz, was then added to established Pex cell infections, treatment was allowed to proceed over a 24-h period, and gonococcal viability was determined, as outlined above. Controls comprised the omission of Cz or the inclusion of 0.1% DMSO (vehicle control) or 0.5 μg/mL (902 nM) ceftriaxone. Assays were performed on three separate occasions.

For all Pex cell infection studies, a nonparametric ANOVA was used to determine the statistical significance of bacterial survival (GraphPad version 8.2.0 for MacOS, GraphPad Software).

### Modified microdilution assays.

To determine whether Cz in vaginal secretions had a direct effect on gonococcal viability, modified microdilution assays were performed using N. gonorrhoeae strain WHO Z. Pooled vaginal fluid from control participants (CBP03-05) was added to select wells of a 384-well plate. Cz (100 μM) was added to the first well after which Cz-containing vaginal fluid was serially diluted 10-fold (as above), yielding a dose range of 10 nM to 100 μM (2.4 ng/mL to 23 μg/mL), with the last well corresponding to a no Cz control. Gonococci were added to each well at a culture density of 10^7^ bacterial per milliliter. Microtiter plates were incubated (3% O_2_, 37°C) for 1 h or 24 h. At each time point, serial dilutions of the vaginal fluid-gonococcal suspension were plated. Viable gonococci were enumerated by counting CFU after a 48-h incubation (37°C, 5% CO_2_). The percentage of viable N. gonorrhoeae was determined as a function of the inoculum. Additional assay controls included 0.1% DMSO (vehicle), 0.5 μg/mL (902 nM) ceftriaxone, 100 μg/mL (209 μM) gentamicin, and an untreated-uninfected, vaginal fluid sterility control. Assays were performed on three separate occasions. Statistical significance of data obtained was determined using a paired Student's *t* test (GraphPad).
