# Supplementary material for: Repurposing Carbamazepine To Treat Gonococcal Infection in Women: Oral Delivery for Control of Epilepsy Generates Therapeutically Effective Levels in Vaginal Secretions
Source: Antimicrob Agents Chemother. 2023 Jan 5;67(1):e00968-22. doi: 10.1128/aac.00968-22 (PMC9872610; doi:10.1128/aac.00968-22)
Supplement: Supplemental file 1 — Supplemental material. Download aac.00968-22-s0001.pdf, PDF file, 0.6 MB [file aac.00968-22-s0001.pdf]

<u>**Supplementary Data**</u>

Table S1. Assessment of the accuracy of the competitive ELISA to measure serum Cz levels compared to the commercial pathology service (CP). Accuracy was determined by dividing the mean serum Cz level determined by the in-house competitive ELISA by the mean serum Cz level determined by the immunoassay performed by the CP and multiplying by 100.

**Participant number**	**Serum Cz by CP**	**Serum CZ by ELISA**	**Accuracy (%)**
CBP08	11.3 µg/ml	9.91 µg/ml	88.7%
CBP12	11.95 µg/ml	11.35 µg/ml	95.0%
CBP13	7.8 µg/ml	7.59 µg/ml	97.3%
CBP14	7.15 µg/ml	6.79 µg/ml	94.7%
CBP15	9.2 µg/ml	12.24 µg/ml	133%
CBP16	11.35 µg/ml	11.68 µg/ml	103%
		**Overall accuracy**	**102%**

Table S2. The effect of vaginal fluid and Cz in vaginal fluid on gonococcal viability.

**Specimen dilution**	**Mean Percent Survival *vs.* Inoculum**				**Reduction (%) in Ng* Viability *vs.* TCM**		
	**1:1 (10%)**	**1:2 (5%)**	**1:3 (3.3%)**	**TCM**	**1:1**	**1:2**	**1:3**
**Control Group**							
**CBP02**<br>n=3	516.85<br>±18.85<br>0.0001^{T}	555.32<br>±17.26<br>0.0001^{UD}<br>0.0001^{T}	609.15<br>±0.92<br>0.0001^{UD}<br>0.0001^{T}	622.70<br>±0.02<br>0.0001^{UD}	17.0<br>±2.94	10.84<br>±1.77	2.18<br>±1.04
**CBP04**<br>n=3	517.71<br>±6.02<br>0.0001^{T}	536.43<br>±13.36<br>0.0196^{UD}<br>0.0001^{T}	597.38<br>±6.47<br>0.0001^{UD}<br>0.0023^{T}	610.42<br>±6.39<br>0.0001^{UD}	15.18<br>±1.12	12.09<br>±3.11	2.12<br>±1.57
**CBP06**<br>n=1	532.68	549.02	575.16	607.84	12.37	9.68	5.38
**CBP07**<br>n=2	527.30<br>±1.16<br>0.0001^{T}	523.59<br>±1.16<br>1.000^{UD}<br>0.0001^{T}	588.35<br>±1.16<br>0.0007^{UD}<br>0.5458^{T}	527.30<br>±1.16<br>0.0007^{UD}	14.92<br>±0.11	15.53<br>±2.46	4.79<br>±4.73
**CBP11**<br>n=1	490.1961	522.8758	571.8954	617.6471	20.63	15.34	16.15
**Test Group**							
**CBP08**<br>n=1	0.14	0.14	0.20	627.45	99.98	99.98	99.97
**CBP12**<br>n=3	0.07<br>±0.02<br>0.0001^{T}	0.10<br>±0.01<br>0.0001^{UD}<br>0.0001^{T}	0.22<br>±0.03<br>0.0001^{UD}<br>0.0001^{T}	610.60<br>±5.21<br>0.0001^{UD}	99.99<br>±0.00	99.98<br>±0.01	99.96<br>±0.01
**CBP13**<br>n=2	0.07<br>±0.01<br>0.0001^{T}	0.09<br>±0.01<br>0.0001^{UD}<br>0.0001^{T}	0.12<br>±0.01<br>0.0001^{UD}<br>0.0001^{T}	625.50<br>±4.58<br>0.0001^{UD}	99.99<br>±0.00	99.99<br>±0.00	99.98<br>±0.00
**CBP14**<br>n=2	0.07<br>±0.01<br>0.0001^{T}	0.07<br>±0.01<br>0.0001^{UD}<br>0.0001^{T}	0.13<br>±0.01<br>0.0001^{UD}<br>0.0001^{T}	625.10<br>±0.92<br>0.0001^{UD}	99.99<br>±0.00	99.99<br>±0.00	99.98<br>±0.00
**CBP15**<br>n=3	0.07<br>±0.02<br>0.0001^{T}	0.08<br>±0.01<br>0.0001^{UD}<br>0.0001^{T}	0.12<br>±0.01<br>0.0001^{UD}<br>0.0001^{T}	623.80<br>±1.98<br>0.0001^{UD}	99.99<br>±0.00	99.99<br>±0.00	99.98<br>±0.00
**CBP16**<br>n=3	0.05<br>±0.01<br>0.0001^{T}	0.08<br>±0.01<br>0.0001^{UD}<br>0.0001^{T}	0.12<br>±0.01<br>0.0001^{UD}<br>0.0001^{T}	591.55<br>±19.21<br>0.0001^{UD}	99.99<br>±0.00	99.99<br>±0.01	99.98<br>±0.00

* Ng – *Neisseria gonorrhoeae*
Specimen dilution: Vaginal fluid was diluted as noted in tissue culture medium (TCM)
TCM: experiments were performed in tissue culture medium rather than vaginal fluid
UD: *p*-values vs data obtained from the use of undiluted vaginal specimens
T: *p*-values vs data obtained from the use of tissue culture medium

Table S3. Inter- and intra-assay precision for the competitive ELISA. A) The inter-assay (within) precision of the competitive ELISA for the 3 matrices (serum, saliva and vaginal fluid) was determined by calculating the coefficient of variation (CoV) for triplicates of each of the standards used in the standard curve. The CoV was calculated by dividing the SD for the 3 replicates by the mean, then multiplying by 100. B) The intra-assay (between) precision was determined for saliva and vaginal fluid assays by calculating the CoV for 6 replicates for each standard used in the standard curve across 2 independent assays. R = replicate.

A)

**Cz conc. (ng/ml)**	**R1**	**R2**	**R3**	**Mean**	**SD**	**CoV (%)**
**Serum**						
50	0.0719	0.0743	0.0724	0.0729	0.0013	**1.7377**
25	0.0900	0.0980	0.0936	0.0939	0.0040	**4.2685**
12.5	0.1305	0.1304	0.1373	0.1327	0.0040	**2.9798**
6.25	0.2004	0.1939	0.2063	0.2002	0.0062	**3.0981**
3.125	0.2984	0.2946	0.3054	0.2995	0.0055	**1.8294**
1.5625	0.4560	0.4608	0.4762	0.4643	0.0106	**2.2728**
0.7813	0.6954	0.6714	0.6939	0.6869	0.0134	**1.9572**
0.3906	1.0576	1.0871	1.0298	1.0582	0.0287	**2.7079**
0.1953	1.4173	1.3237	1.3892	1.3767	0.0480	**3.4886**
0.0977	1.8813	1.8254	1.7537	1.8201	0.0640	**3.5142**
0.0488	2.0017	2.0763	2.0356	2.0379	0.0374	**1.8329**
0.0000	2.4173	2.5218	2.3790	2.4394	0.0739	**3.0300**
**Saliva**						
50.0000	0.0691	0.0702	0.0720	0.0704	0.0015	**2.0786**
25.0000	0.0853	0.0873	0.0915	0.0880	0.0032	**3.5945**
12.5000	0.1249	0.1225	0.1238	0.1237	0.0012	**0.9709**
6.2500	0.1834	0.1757	0.1805	0.1799	0.0039	**2.1621**
3.1250	0.3025	0.2723	0.2835	0.2861	0.0153	**5.3362**
1.5625	0.4914	0.4251	0.4341	0.4502	0.0360	**7.9882**
0.7813	0.6958	0.6609	0.6487	0.6685	0.0244	**3.6568**
0.3906	0.9997	0.9491	0.9225	0.9571	0.0392	**4.0975**
0.1953	1.3704	1.2785	1.3151	1.3213	0.0463	**3.5015**
0.0977	1.7380	1.6673	1.6687	1.6913	0.0404	**2.3899**
0.0488	1.8591	1.9144	1.8769	1.8835	0.0282	**1.4988**
0.0000	2.2011	2.2605	2.1875	2.2164	0.0388	**1.7516**
**Vaginal fluid**						
50.0000	0.0739	0.0670	0.0680	0.0696	0.0037	**5.3548**
25.0000	0.0860	0.0882	0.0888	0.0877	0.0015	**1.6816**
12.5000	0.1207	0.1185	0.1231	0.1208	0.0023	**1.9051**
6.2500	0.1815	0.1766	0.1895	0.1825	0.0065	**3.5674**
3.1250	0.2624	0.2733	0.2721	0.2693	0.0060	**2.2197**
1.5625	0.3744	0.3767	0.3983	0.3831	0.0132	**3.4414**
0.7813	0.6161	0.6134	0.6326	0.6207	0.0104	**1.6745**
0.3906	0.9138	0.9343	0.9537	0.9339	0.0200	**2.1364**
0.1953	1.2527	1.2659	1.3087	1.2758	0.0293	**2.2947**
0.0977	1.5482	1.6070	1.6342	1.5965	0.0440	**2.7534**
0.0488	1.7584	1.8424	1.8445	1.8151	0.0491	**2.7059**

0.0000	2.1022	2.1178	2.2912	2.1704	0.1049	**4.8335**

B)

**Cz conc. (ng/ml)**	**R1**	**R2**	**R3**	**R4**	**R5**	**R6**	**Mean**	**SD**	**CoV**
**Saliva**									
50.0000	0.0691	0.0702	0.0720	0.0683	0.0782	0.0742	0.0720	0.0037	**5.1453**
25.0000	0.0853	0.0873	0.0915	0.0929	0.1005	0.0959	0.0922	0.0056	**6.0400**
12.5000	0.1249	0.1225	0.1238	0.1388	0.1390	0.1270	0.1293	0.0076	**5.8423**
6.2500	0.1834	0.1757	0.1805	0.2154	0.2084	0.1981	0.1936	0.0162	**8.3633**
3.1250	0.3025	0.2723	0.2835	0.3577	0.3240	0.2943	0.3057	0.0309	**10.1216**
1.5625	0.4914	0.4251	0.4341	0.5711	0.5200	0.4716	0.4855	0.0548	**11.2939**
0.7813	0.6958	0.6609	0.6487	0.7658	0.7486	0.7217	0.7069	0.0470	**6.6512**
0.3906	0.9997	0.9491	0.9225	1.1561	1.0698	1.0742	1.0286	0.0877	**8.5252**
0.1953	1.3704	1.2785	1.3151	1.3860	1.3907	1.3479	1.3481	0.0440	**3.2641**
0.0977	1.7380	1.6673	1.6687	1.7271	1.7247	1.6939	1.7033	0.0310	**1.8210**
0.0488	1.8591	1.9144	1.8769	2.0416	1.9910	1.9931	1.9460	0.0731	**3.7557**
0.0000	2.2011	2.2605	2.1875	2.3275	2.3558	2.3355	2.2780	0.0724	**3.1789**
**Vaginal fluid**									
50.0000	0.0739	0.0670	0.0680	0.0804	0.0759	0.0791	0.0740	0.0056	**7.5329**
25.0000	0.0860	0.0882	0.0888	0.0974	0.0916	0.1046	0.0928	0.0070	**7.5494**
12.5000	0.1207	0.1185	0.1231	0.1358	0.1366	0.1331	0.1280	0.0081	**6.3327**
6.2500	0.1815	0.1766	0.1895	0.2054	0.2091	0.2085	0.1951	0.0144	**7.3930**
3.1250	0.2624	0.2733	0.2721	0.3094	0.3210	0.3179	0.2927	0.0262	**8.9535**
1.5625	0.3744	0.3767	0.3983	0.4538	0.4619	0.4769	0.4237	0.0458	**10.8062**
0.7813	0.6161	0.6134	0.6326	0.7048	0.7095	0.6782	0.6591	0.0439	**6.6596**
0.3906	0.9138	0.9343	0.9537	1.0441	1.0719	1.1143	1.0054	0.0823	**8.1900**
0.1953	1.2527	1.2659	1.3087	1.3687	1.3857	1.3873	1.3282	0.0607	**4.5676**
0.0977	1.5482	1.6070	1.6342	1.7475	1.7796	1.8846	1.7002	0.1255	**7.3787**
0.0488	1.7584	1.8424	1.8445	2.0979	2.0747	2.0977	1.9526	0.1540	**7.8882**
0.0000	2.1022	2.1178	2.2912	2.3457	2.4351	2.3900	2.2803	0.1404	**6.1557**

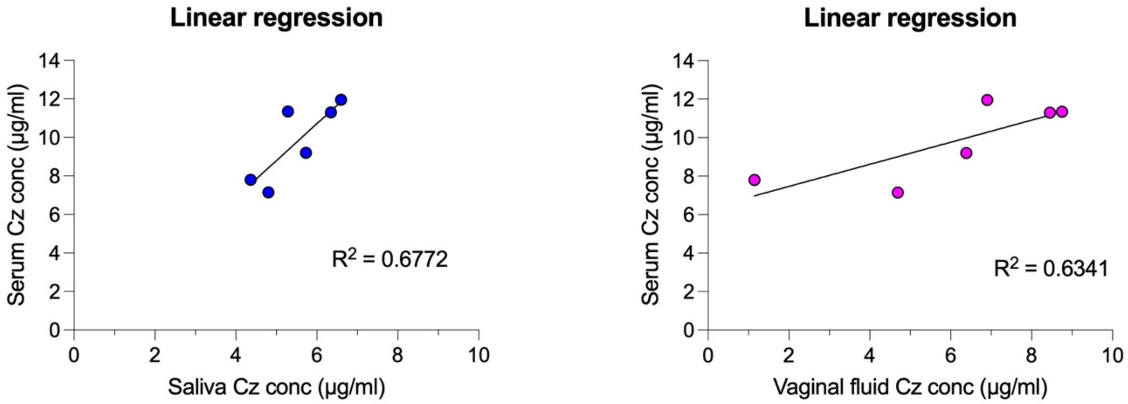

Figure S1. Linear regression analysis of (A) saliva and (B) vaginal fluid Cz concentrations determined via the competitive ELISA and serum Cz concentrations determined via the commercial pathology service for subjects taking Cz (test subjects only; n = 6). Linear regression analysis was performed using Graphpad Prism 9.

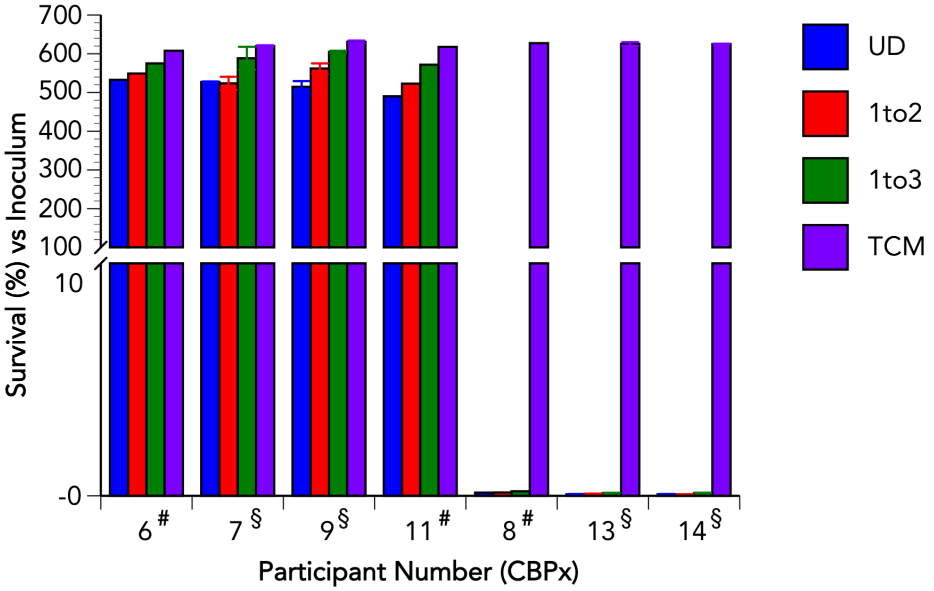

Figure S2. Carbamazepine is present in vaginal fluid at concentrations sufficient to effectively treat gonococcal infection. The effect of Cz on *N. gonorrhoeae* strain WHO Z survival during a Pex cell infection was examined using vaginal fluid from participants (CBP) whom were either not taking (participants CBP06, 07, 09, 11) or taking (participants CBP08, 13, and 14) Cz for medically indicated reasons not related to the present study. Values given are the mean (variance) of the percentage of viable gonococci recovered from Pex cells at 24h post-infection versus the infection inocula. Data are shown for those study participants, in which the volume of available vaginal fluid was limited and only allowed for an n of 1 (#) or an n of 2 (§), as noted on the x-axis. Experiments were performed using undiluted (UD; *i.e.,* 10%, see text) vaginal fluid, vaginal fluid that was diluted 1:2 (5%) or 1:3 (3.3%) in tissue culture medium, or in tissue culture medium (TCM), as noted. The percent reduction in the number of viable gonococci recovered from Pex cell infections using the vaginal fluid from each participant is provided in Table S2.

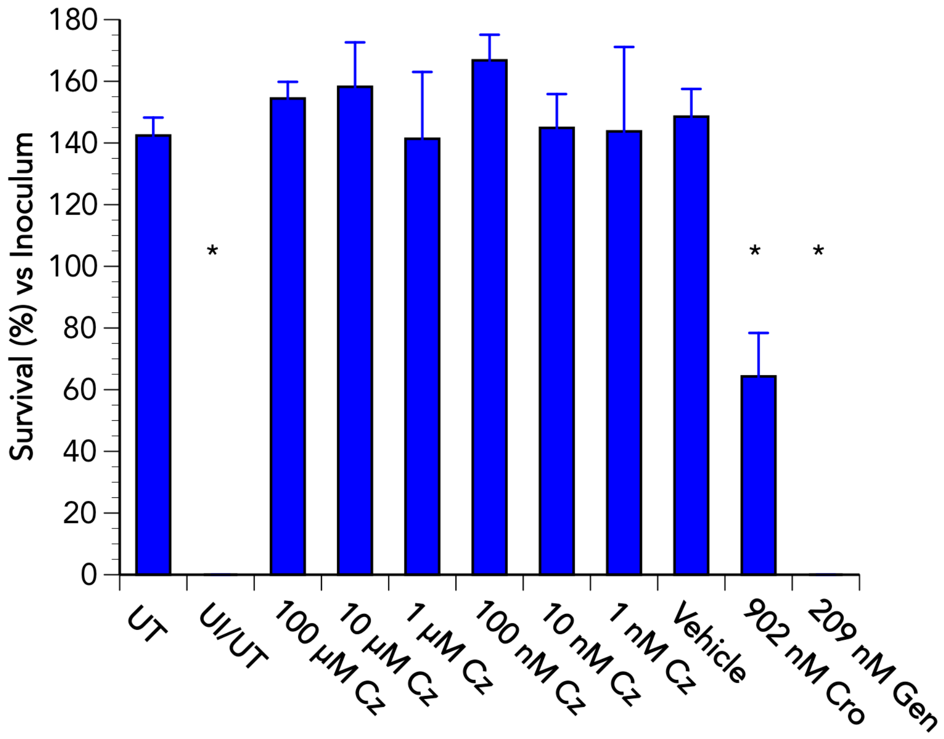

Figure S3. Carbamazepine is not effective against *N. gonorrhoeae* in the absence of Pex cells. The effect of Cz on *N. gonorrhoeae* strain WHO Z in the absence of Pex cells was examined using pooled vaginal fluid from participants whom were not taking Cz (participants CBP03, 04, 05, see Table 1). Values given are the mean (variance) of the percentage of viable gonococci recovered at 1h post-treatment versus the infection inocula. Assays were performed on 3 separate occasions. UT – untreated, gonococci incubated in vaginal fluid only, UI/UT – uninfected/untreated, vaginal fluid that was not inoculated with gonococci or drug, Vehicle – 0.1% DMSO, Cro – ceftriaxone, Gen – gentamicin. * $p \leq 0.032$
